# Supplementary material for: Classifying atopic dermatitis: protocol for a systematic review of subtypes (phenotypes) and associated characteristics
Source: BMJ Open. 2018 Sep 17;8(9):e023097. doi: 10.1136/bmjopen-2018-023097 (PMC6144401; doi:10.1136/bmjopen-2018-023097)
Supplement: Author's manuscript [file bmjopen-2018-023097.draft_revisions.pdf]

BMJ Open is committed to open peer review. As part of this commitment we make the peer review history of every article we publish publicly available.

When an article is published we post the peer reviewers' comments and the authors' responses online. We also post the versions of the paper that were used during peer review. These are the versions that the peer review comments apply to.

The versions of the paper that follow are the versions that were submitted during the peer review process. They are not the versions of record or the final published versions. They should not be cited or distributed as the published version of this manuscript.

BMJ Open is an open access journal and the full, final, typeset and author-corrected version of record of the manuscript is available on our site with no access controls, subscription charges or pay-per-view fees (http://bmjopen.bmj.com).

If you have any questions on BMJ Open's open peer review process please email info.bmjopen@bmj.com

BMJ Open

Classifying eczema: protocol for a systematic review of eczema subtypes (phenotypes) and associated characteristics

Journal:	*BMJ Open*
Manuscript ID	bmjopen-2018-023097
Article Type:	Protocol
Date Submitted by the Author:	20-Mar-2018
Complete List of Authors:	Mulick, Amy; London School of Hygiene and Tropical Medicine, ; London School of Hygiene and Tropical Medicine<br>Allen, Victoria; London School of Hygiene and Tropical Medicine, Non-Communicable Disease Epidemiology<br>Williams, HC; University of Nottingham School of Medicine, Centre of Evidence Based Dermatology<br>Grindlay, Douglas; University of Nottingham School of Medicine, Centre of Evidence Based Dermatology<br>Pearce, Neil; London School of Hygiene and Tropical Medicine, Medical Statistics<br>Abuabara, Katrina; UCSF School of Medicine, Dermatology<br>Langan, Sinead; London School of Hygiene and Tropical Medicine, Non-Communicable Disease Epidemiology
Keywords:	Eczema < DERMATOLOGY, atopic dermatitis, phenotype, classification

SCHOLARONE™
Manuscripts

For peer review only

Classifying eczema: protocol for a systematic review of eczema subtypes (phenotypes) and associated characteristics

Authors: Amy Mulick[1*], Victoria Allen[1], Hywel C Williams[2], Douglas JC Grindlay[2], Neil Pearce[1,3], Katrina Abuabara[4], Sinéad M Langan[1]

1 Department of Non-Communicable Disease Epidemiology, London School of Hygiene and Tropical Medicine, London, UK

2 Centre of Evidence Based Dermatology, University of Nottingham, Nottingham, UK

3 Department of Medical Statistics, London School of Hygiene and Tropical Medicine, London, UK

4 Program for Clinical Research, Department of Dermatology, UCSF School of Medicine, University of California San Francisco, San Francisco, CA, USA

Email: Amy Mulick amy.mulick@lshtm.ac.uk, Victoria Allen victoria.b.c.allen@gmail.com, Hywel Williams Hywel.Williams@nottingham.ac.uk, Douglas Grindlay Douglas.Grindlay@nottingham.ac.uk, Neil Pearce neil.pearce@lshtm.ac.uk, Katrina Abuabara katrina.abuabara@ucsf.edu, Sinéad Langan sinead.langan@lshtm.ac.uk

*corresponding author: amy.mulick@lshtm.ac.uk, Department of Non-Communicable Disease Epidemiology, London School of Hygiene and Tropical Medicine, Keppel Street, London WC1E 7HT, UK

For peer review only

For peer review only - http://bmjopen.bmj.com/site/about/guidelines.xhtml

Abstract

Introduction

Eczema is a complex disease with differing clinical presentations. Many attempts have been made to identify uniform subtypes, or phenotypes, of eczema in order to identify different aetiologies, estimate more accurate clinical prognoses or predict treatment efficacy. However, no consensus yet exists on exactly what defines these phenotypes or how many there are and whether they are genuine or statistical artefacts. This review aims to identify previously reported eczema phenotypes, the features used to define them and any characteristics or clinical outcomes significantly associated with them.

Methods and analysis

We will search Ovid Embase, Ovid MEDLINE, Scopus, and Web of Science from inception to 5 February 2018 for studies attempting to classify eczema in humans using any cross-sectional or longitudinal epidemiological or interventional design. Primary outcomes are eczema phenotypes, features used to define them and characteristics associated with them in subsequent analyses. A secondary outcome is the methodological approach used to derive them. Two reviewers will independently screen titles and abstracts for inclusion, extract data and assess study quality. We will present the results of this review descriptively and with frequencies where possible.

Ethics and dissemination

Ethical approval is not required for this study as it is a systematic review. We will report results from this systematic review in a peer-reviewed journal. The main value of this study will be to inform further research.

PROSPERO registration number

CRD42018087500

For peer review only

For peer review only - http://bmjopen.bmj.com/site/about/guidelines.xhtml

Article Summary

Strengths and limitations of this study

- Prospectively registered review reported consistent with PRISMA guidelines
- This study will provide the first comprehensive review of current evidence on phenotypic subgroupings of eczema. This is an important topic in a time of rapid development of new therapeutic options for eczema
- It will be difficult to assess bias resulting from non-publication of studies or selective reporting of results
- It may be difficult to synthesize the results, due to expected heterogeneity in identified phenotypes and in the outcomes/other characteristics explored

For peer review only

For peer review only - http://bmjopen.bmj.com/site/about/guidelines.xhtml

Introduction

Eczema, also known as atopic eczema, atopic dermatitis, neurodermatitis and Besnier's prurigo[13], is a complex disease with variable clinical presentations, associations with other atopic diseases and clinical courses.[1] Traditionally eczema was characterised as an allergic disease of childhood, but it is now well-established that non-allergic forms exist and patients have been subdivided into those with and without atopy. However, there is much evidence suggesting that this dichotomisation is not clinically useful;[2] despite the use of names such as "atopic dermatitis" or "atopic eczema", up to two thirds of patients with eczema are not atopic and atopy status does not predict outcomes or treatment responses.[3] Furthermore, genetic research in the last decade has led to a paradigm shift in understanding the aetiology of eczema, from being considered primarily an allergic/immunological disorder to understanding the additional importance of skin barrier dysfunction.[4, 5]

A phenotype is a set of observable characteristics of an individual resulting from the interaction of its genotype with the environment.[6] This includes, for example, the individual's appearance, development, and behaviour. In an epidemiological context the word phenotype additionally refers to subtypes of a disease that are defined by different phenotypic appearances. These subtypes may be referred to using a variety of terminology in addition to 'phenotypes': for example, they may be called disease classifications, subgroups, typologies, strata, patterns or taxonomies by different researchers. Subtypes or phenotypes are not to be confused with endotypes, which are sub-types of a disease defined specifically by different functional or pathobiological mechanisms. One endotype could give rise to multiple phenotypes.

Current eczema phenotypes are based on clinical (exogenous) features, genetic or immunological (endogenous) features, comorbidities or symptom course. In general, study participants have been characterised by the presence or absence of a particular characteristic, e.g. history of eczema herpeticum (sometimes called an exophenotype), elevated serum IgE and low filaggrin protein expression (endophenotypes). Clinical comorbidities (such as asthma and hay fever or ichthyosis) and symptom trajectories have also been used to define phenotypes. [7-10] Recent studies have highlighted the heterogeneity of eczema symptom trajectories and have suggested that different disease clinical courses in eczema are associated with immunological characteristics and disease locations.[11]

If there exist a number of eczema phenotypes, each of which exhibits homogeneous disease characteristics, this has the potential to inform eczema aetiology (i.e. identify endotypes), patient prognosis and prediction of treatment efficacy. Finding simple, meaningful disease phenotypes can elucidate the different biological pathways underpinning them, leading to the discovery of endotypes (aetiology); knowledge of the markers for these pathways can assist clinicians in diagnosing and managing patients' symptoms more accurately, particularly on whether to expect persistence or remission (prognosis) and whether a potential treatment will work for them (prediction). At present, treatment for long-term eczema is not based on disease phenotypes; treatment is symptomatic and may have associated toxicity.[12] The identification of meaningful phenotypes has potential to improve treatment strategies and provide biological insights into the future development of new ones.

No study has systematically reviewed the literature summarizing the currently existing eczema phenotypic classifications. We will do this, aiming to identify previously reported eczema phenotypes in studies specifically designed to identify subtypes of eczema. We will also describe the

For peer review only

features used to define them and whether they were predictive of or associated with any outcomes, concurrent conditions, treatment response or other relevant variables.

Methods and analysis

Eligibility criteria

We will search for studies with any cross-sectional or longitudinal epidemiological or interventional design whose primary or secondary aim is to define or identify subtypes/classifications/phenotypes of eczema in humans of any age and gender. Features used to identify phenotypes could be based on either static or dynamic characteristics of their populations. We expect that most studies will contain only individuals with eczema but some studies may have included individuals without eczema, for example as a negative control group[10] or because an eczema diagnosis was unavailable. We will include these studies. If included, the control population would be people known to be free of eczema or who have a low probability of having eczema, including people who may have asthma, hay fever and other atopic diseases.

We will exclude: studies of localized eczema such as hand eczema and other types of eczema such as contact dermatitis and adult seborrheic dermatitis (for studies prior to 1990 we may include seborrheic dermatitis of infancy); literature reviews, books, book chapters, case reports, case series and in-progress phenotyping studies (abstracts), but not ongoing birth or other cohort studies; and conference proceedings and abstracts, as they are unlikely to provide sufficient detail on the definitions of eczema phenotypes.

Information sources

We will search Ovid Embase, Ovid MEDLINE, Scopus, and Web of Science from inception to 5 February 2018 for publications in any language using English search terms. We will limit results to human studies published in original journal articles (published or in press, excluding retracted articles) or systematic reviews. We will interrogate the reference lists from the most recent two review articles in each database.

Search strategy

We will use conduct the following MEDLINE search:

((exp phenotype/ OR classif* OR sub?type OR phenotyp* OR taxonomy OR heterogeneity OR complexity OR pattern OR patterns OR disease type* OR disease typolog* OR *stratif* OR strata) AND (exp dermatitis, atopic/ OR exp eczema/ OR exp neurodermatitis/ OR eczema* OR atopic dermatitis OR neurodermatitis OR besnier* prurigo))

Searches for the other databases will be matched as closely as possible to this using appropriate syntax and headings.

Study Records

Data management

Literature search results will be uploaded to Covidence web software, which will be used for all stages of the review process including title/abstract screening, full-text screening, data extraction, bias/quality assessment and process flow capture. We will develop and test screening questions

For peer review only - http://bmjopen.bmj.com/site/about/guidelines.xhtml

based on the inclusion and exclusion criteria and a data extraction form based on the outcomes and pilot them on a subset of studies.

Selection process

Two reviewers will scan all titles and abstracts independently. Publications that both reviewers record as 'not relevant' will not be retrieved for full-text review; the full text of all others will be retrieved into a 'short-list'.

Data collection process

During the short-list review, data from the texts in full will be extracted by two reviewers using a pre-designed data extraction form and disagreements will be resolved by discussion among investigators.

Publications will be automatically included in this study if both reviewers independently assess them as meeting the inclusion criteria and excluded from this study where both assess them as not meeting the criteria. Disagreements will be resolved through referral to a third reviewer.

Data Items

We will extract three domains of data from publications included in our study. In the event of subgroup analyses, such as studies reporting different eczema phenotypes for males and females, we will extract data from the from the combined-group phenotypes if available. If unavailable, we will extract data for each phenotype separately. We believe it unlikely that more than one subgroup analysis will have been conducted in the absence of a main single analysis, but if this happens we will extract data from the first subgroups reported in the Results section of the paper.

Study data

Design, year(s) conducted, country/countries conducted in, setting conducted in (population-based, specialist etc.), inclusion/exclusion criteria, number of participants, age range of participants, gender balance of participants, notable comorbidities (from study design) including, e.g., proportions with other atopic diseases.

Eczema data

Definition codes/criteria, severity definition, prevalence and incidence (if relevant).

Outcomes

A qualitative description of the phenotypes, features (variables) used to define phenotypes, age at the time of phenotype definition, proportion of individuals in each phenotype, qualitative description of any variables statistically significantly associated (in subsequent analyses) with the phenotypes (we will not report the effect estimates), statistical or other method used for classification, whether controls were used in classification algorithm.

Outcomes and prioritisation

The outcomes for this review are eczema phenotypes and associated characteristics, all of which are qualitative (i.e. nonnumeric) data.

Primary outcomes are:

1) eczema phenotypes reported in published papers;

For peer review only - http://bmjopen.bmj.com/site/about/guidelines.xhtml

For peer review only

2) the features used to define the phenotypes (i.e. the 'exposure' variables); and
3) the characteristics statistically significantly associated (in subsequent analyses) with the phenotypes, if any, which could include: long-term clinical outcomes such as disease persistence or severity; concurrent conditions such as other atopic disease; treatment response; genetic data (e.g. *FLG* mutations); or any other variables reported in studies.

Our secondary outcome is:

1. a brief summary of the methodological approaches used to derive eczema phenotypes.

We will not proactively seek particular values of the outcomes described above; we will collect any and all outcomes reported in the publications.

Risk of bias

We will assess quality within and between studies using a checklist modified from the GRADE tool[14] for clinical trials or observational studies. Where relevant, instead of effect estimates and confidence intervals, we will assess equivalent parameters for the methods used to derive subtypes. We will additionally consider upgrading the final assessment by one level if the study is prospective, population-based or has been replicated.

Within studies

We will treat the exposure as the feature(s) used to identify phenotypes, which will vary between studies, and the outcome as the identified phenotypes. We will give an initial rating of 'high quality' for randomized controlled trials or 'low quality' for observational studies and then judge risk of bias based on the extracted information on each of the domains relevant to the study type. Each item will be rated 'high risk of bias' or 'low risk of bias' and will be synthesized according to GRADE recommendations. Based on the synthesized judgement we will consider whether to downgrade or upgrade the initial quality assessment. Final possible quality assessments for individual studies are 'high', 'moderate', 'low' and 'very low'.

Between studies

We anticipate the phenotype descriptions to vary between studies according to the features used to define them (exposure variables), so for a given outcome it may not be possible to synthesize across studies. However, where it is possible we will synthesize study results according to GRADE recommendations. Final possible quality assessments for the body of evidence are 'high', 'moderate' and 'low'

Data Synthesis

The nature of the outcomes for this review preclude quantitative synthesis, so we will report our findings narratively. Our primary outcomes (eczema phenotypes, the features used to define them and the outcomes/characteristics associated with them) are all qualitative and we expect them to differ between studies, but where possible we will group them into sensible categories and report frequencies.

For our secondary outcome we will report the type and frequency of methodological approach used to derive the phenotypes.

We expect heterogeneity in all our outcomes because we have no reason to expect studies will have used similar protocols to explore phenotypes: for example, studies will probably have used different exposure variables to define phenotypes and will represent populations of people with different

For peer review only

characteristics. To explore this we will compare the: participant age and gender balance, study design, World Health Organization (WHO) region (Africa, Americas, Southeast Asia, Europe, Eastern Mediterranean and Western Pacific), and eczema definition for each study, where possible. We will also look at hospital-based and population-based studies separately.

Meta-biases

Outcomes in this review may be prone to meta-bias resulting from an absence of studies looking at important indicators of phenotypes, non-publication of study results or selective reporting of outcomes. For our qualitative outcomes, it will not be possible to measure this objectively. We will speculate on whether this is likely to be an important limiting factor in interpreting our results.

Ethics and dissemination

This systematic review protocol was registered with the International Prospective Register of Systematic Reviews (PROSPERO) on 31 January 2018 and was last updated on 12 March 2018. Any amendments to the protocol will be documented on the PROSPERO site contemporaneously, with full explanation of any change. Ethical approval is not required for this study as it is a systematic review. We plan to submit a report of our findings for publication in a peer-reviewed journal. Findings from the report will also support the first author's PhD thesis.

For peer review only

For peer review only - http://bmjopen.bmj.com/site/about/guidelines.xhtml

Authors' contributions

AM contributed to the design of the study, developed the search strategy, drafted the PROSPERO protocol and this manuscript and is the guarantor. VA contributed to the design of the study. HW, NP and KA provided critical feedback on the PROSPERO protocol and drafts of the manuscript. DG approved the search strategy. SML contributed to the conception and design of the study, and provided critical feedback on the search strategy, PROSPERO protocol and drafts of the manuscript. All authors read, provided feedback and approved the final manuscript.

Funding statement

Publication of this manuscript was funded by a Wellcome Senior Clinical Fellowship to SML (205039/Z/16/Z). AM and SML were supported by this grant. This research has received funding from the European Research Council under the European Union's Seventh Framework Programme (FP7/2007-2013) / ERC grant agreement no 668954.

Role of the funders

These funders provide salary support. They are not involved in any other aspect of the project, such as the design of the project's protocol and analysis plan. They will not be involved in the collection, analysis and interpretation of data; the writing of the report; or the decision to submit the paper for publication.

Competing interests

The authors declare we have no competing interests.

For peer review only

References

1. Leung DY, Guttman-Yassky E. Deciphering the complexities of atopic dermatitis: shifting paradigms in treatment approaches. J Allergy Clin Immunol. 2014 Oct;134(4):769-79
2. Williams H, Flohr C. How epidemiology has challenged 3 prevailing concepts about atopic dermatitis. J Allergy Clin Immunol. 2006 Jul;118(1):209-13
3. Flohr C, Johansson SG, Wahlgren CF, Williams H. How atopic is atopic dermatitis? J Allergy Clin Immunol. 2004 Jul;114(1):150-8
4. Sandilands A, O'Regan G, Liao H, Zhao Y, Terron-Kwiatkowski A, Watson R, et al. Prevalent and rare mutations in the gene encoding filaggrin cause ichthyosis vulgaris and predispose individuals to atopic dermatitis. J Invest Dermatol. 2006 Aug;126(8):1770-5
5. Brown SJ, Kroboth K, Sandilands A, Campbell LE, Pohler E, Kezic S, et al. Intragenic copy number variation within filaggrin contributes to the risk of atopic dermatitis with a dose-dependent effect. J Invest Dermatol. 2012 Jan;132(1):98-104
6. Oxford English Dictionary. 2018. Phenotype.
7. Beck LA, Boguniewicz M, Hata T, Schneider LC, Hanifin J, Gallo R, et al. Phenotype of atopic dermatitis subjects with a history of eczema herpeticum. J Allergy Clin Immunol. 2009 Aug;124(2):260-9, 9 e1-7
8. Brown SJ, Relton CL, Liao H, Zhao Y, Sandilands A, McLean WHI, et al. Filaggrin haploinsufficiency is highly penetrant and is associated with increased severity of eczema: Further delineation of the skin phenotype in a prospective epidemiological study of 792 school children. British Journal of Dermatology. 2009;161(4):884-9
9. Flohr C, England K, Radulovic S, McLean WHI, Campbell LE, Barker J, et al. Filaggrin loss-of-function mutations are associated with early-onset eczema, eczema severity and transepidermal water loss at 3 months of age. British Journal of Dermatology. 1333;163(6):1333-6
10. Paternoster L, Savenije OEM, Heron J, Evans DM, Vonk JM, Brunekreef B, et al. Identification of atopic dermatitis subgroups in children from 2 longitudinal birth cohorts. J Allergy Clin Immunol. 2017 Nov 10
11. Garmhausen D, Hagemann T, Bieber T, Dimitriou I, Fimmers R, Diepgen T, et al. Characterization of different courses of atopic dermatitis in adolescent and adult patients. Allergy. 2013 Apr;68(4):498-506
12. Schmitt J, Schakel K, Schmitt N, Meurer M. Systemic treatment of severe atopic eczema: A systematic review. Acta Dermato Venereologica. 2007;87(2):100-11
13. Williams HC. Atopic Dermatitis: The epidemiology, causes and prevention of atopic eczema: Cambridge University Press; 2000.
14. Guyatt GH, Oxman AD, Vist G, Kunz R, Brozek J, Alonso-Coello P, et al. GRADE guidelines: 4. Rating the quality of evidence--study limitations (risk of bias). J Clin Epidemiol. 2011 Apr;64(4):407-15

For peer review only

For peer review only - http://bmjopen.bmj.com/site/about/guidelines.xhtml

PRISMA-P (Preferred Reporting Items for Systematic review and Meta-Analysis Protocols) 2015 checklist: recommended items to address in a systematic review protocol*

Section and topic	Item No	Checklist item	Reported on page #
**ADMINISTRATIVE INFORMATION**			
Title:			
Identification	1a	Identify the report as a protocol of a systematic review	1
Update	1b	If the protocol is for an update of a previous systematic review, identify as such	n/a
Registration	2	If registered, provide the name of the registry (such as PROSPERO) and registration number	2,8
Authors:			
Contact	3a	Provide name, institutional affiliation, e-mail address of all protocol authors; provide physical mailing address of corresponding author	1
Contributions	3b	Describe contributions of protocol authors and identify the guarantor of the review	9
Amendments	4	If the protocol represents an amendment of a previously completed or published protocol, identify as such and list changes; otherwise, state plan for documenting important protocol amendments	8
Support:			
Sources	5a	Indicate sources of financial or other support for the review	9
Sponsor	5b	Provide name for the review funder and/or sponsor	9
Role of sponsor or funder	5c	Describe roles of funder(s), sponsor(s), and/or institution(s), if any, in developing the protocol	9
**INTRODUCTION**			
Rationale	6	Describe the rationale for the review in the context of what is already known	4
Objectives	7	Provide an explicit statement of the question(s) the review will address with reference to participants, interventions, comparators, and outcomes (PICO)	4-5
**METHODS**			
Eligibility criteria	8	Specify the study characteristics (such as PICO, study design, setting, time frame) and report characteristics (such as years considered, language, publication status) to be used as criteria for eligibility for the review	5
Information sources	9	Describe all intended information sources (such as electronic databases, contact with study authors, trial registers or other grey literature sources) with planned dates of coverage	5
Search strategy	10	Present draft of search strategy to be used for at least one electronic database, including planned limits, such that it could be repeated	5

For peer review only

Study records:			
Data management	11a	Describe the mechanism(s) that will be used to manage records and data throughout the review	5-6
Selection process	11b	State the process that will be used for selecting studies (such as two independent reviewers) through each phase of the review (that is, screening, eligibility and inclusion in meta-analysis)	6
Data collection process	11c	Describe planned method of extracting data from reports (such as piloting forms, done independently, in duplicate), any processes for obtaining and confirming data from investigators	6
Data items	12	List and define all variables for which data will be sought (such as PICO items, funding sources), any pre-planned data assumptions and simplifications	6
Outcomes and prioritization	13	List and define all outcomes for which data will be sought, including prioritization of main and additional outcomes, with rationale	6-7
Risk of bias in individual studies	14	Describe anticipated methods for assessing risk of bias of individual studies, including whether this will be done at the outcome or study level, or both; state how this information will be used in data synthesis	7
Data synthesis	15a	Describe criteria under which study data will be quantitatively synthesised	7-8
	15b	If data are appropriate for quantitative synthesis, describe planned summary measures, methods of handling data and methods of combining data from studies, including any planned exploration of consistency (such as I^2, Kendall's τ)	n/a
	15c	Describe any proposed additional analyses (such as sensitivity or subgroup analyses, meta-regression)	7-8
	15d	If quantitative synthesis is not appropriate, describe the type of summary planned	7-8
Meta-bias(es)	16	Specify any planned assessment of meta-bias(es) (such as publication bias across studies, selective reporting within studies)	8
Confidence in cumulative evidence	17	Describe how the strength of the body of evidence will be assessed (such as GRADE)	7

*** It is strongly recommended that this checklist be read in conjunction with the PRISMA-P Explanation and Elaboration (cite when available) for important clarification on the items. Amendments to a review protocol should be tracked and dated.** **The copyright for PRISMA-P (including checklist) is held by the PRISMA-P Group and is distributed under a Creative Commons Attribution Licence 4.0.**

From: Shamseer L, Moher D, Clarke M, Ghersi D, Liberati A, Petticrew M, Shekelle P, Stewart L, PRISMA-P Group. Preferred reporting items for systematic review and meta-analysis protocols (PRISMA-P) 2015: elaboration and explanation. BMJ. 2015 Jan 2;349(jan02 1):g7647.

For peer review only

BMJ Open

Classifying atopic dermatitis: protocol for a systematic review of subtypes (phenotypes) and associated characteristics

Journal:	*BMJ Open*
Manuscript ID	bmjopen-2018-023097.R1
Article Type:	Protocol
Date Submitted by the Author:	25-Jul-2018
Complete List of Authors:	Mulick, Amy; London School of Hygiene and Tropical Medicine, ; London School of Hygiene and Tropical Medicine<br>Allen, Victoria; London School of Hygiene and Tropical Medicine, Non-Communicable Disease Epidemiology<br>Williams, HC; University of Nottingham School of Medicine, Centre of Evidence Based Dermatology<br>Grindlay, Douglas; University of Nottingham School of Medicine, Centre of Evidence Based Dermatology<br>Pearce, Neil; London School of Hygiene and Tropical Medicine, Medical Statistics<br>Abuabara, Katrina; UCSF School of Medicine, Dermatology<br>Langan, Sinead; London School of Hygiene and Tropical Medicine, Non-Communicable Disease Epidemiology
**Primary Subject Heading**:	Epidemiology
Secondary Subject Heading:	Dermatology
Keywords:	Eczema < DERMATOLOGY, atopic dermatitis, phenotype, classification

SCHOLARONE™
Manuscripts

Classifying atopic dermatitis: protocol for a systematic review of subtypes (phenotypes) and associated characteristics

Authors: Amy Mulick[1*], Victoria Allen[1], Hywel C Williams[2], Douglas JC Grindlay[2], Neil Pearce[1,3], Katrina Abuabara[4], Sinéad M Langan[1]

1 Department of Non-Communicable Disease Epidemiology, London School of Hygiene and Tropical Medicine, London, UK

2 Centre of Evidence Based Dermatology, University of Nottingham, Nottingham, UK

3 Department of Medical Statistics, London School of Hygiene and Tropical Medicine, London, UK

4 Program for Clinical Research, Department of Dermatology, UCSF School of Medicine, University of California San Francisco, San Francisco, CA, USA

Email: Amy Mulick amy.mulick@lshtm.ac.uk, Victoria Allen victoria.b.c.allen@gmail.com, Hywel Williams Hywel.Williams@nottingham.ac.uk, Douglas Grindlay Douglas.Grindlay@nottingham.ac.uk, Neil Pearce neil.pearce@lshtm.ac.uk, Katrina Abuabara katrina.abuabara@ucsf.edu, Sinéad Langan sinead.langan@lshtm.ac.uk

*corresponding author: amy.mulick@lshtm.ac.uk, Department of Non-Communicable Disease Epidemiology, London School of Hygiene and Tropical Medicine, Keppel Street, London WC1E 7HT, UK

For peer review only

For peer review only - http://bmjopen.bmj.com/site/about/guidelines.xhtml

Abstract

Introduction

Atopic dermatitis is a complex disease with differing clinical presentations. Many attempts have been made to identify uniform subtypes, or phenotypes, of atopic dermatitis in order to identify different aetiologies, improve diagnosis, estimate more accurate clinical prognoses, inform treatment/management or predict treatment efficacy/effectiveness. However, no consensus yet exists on exactly what defines these phenotypes or how many there are and whether they are genuine or statistical artefacts. This review aims to identify previously reported phenotypes of atopic dermatitis, the features used to define them and any characteristics or clinical outcomes significantly associated with them.

Methods and analysis

We will search Ovid Embase, Ovid MEDLINE, and Web of Science from inception to the latest available date at the time of the search for studies attempting to classify atopic dermatitis in humans using any cross-sectional or longitudinal epidemiological or interventional design. Primary outcomes are atopic dermatitis phenotypes, features used to define them and characteristics associated with them in subsequent analyses. A secondary outcome is the methodological approach used to derive them. Two reviewers will independently screen titles and abstracts for inclusion, extract data and assess study quality. We will present the results of this review descriptively and with frequencies where possible.

Ethics and dissemination

Ethical approval is not required for this study as it is a systematic review. We will report results from this systematic review in a peer-reviewed journal. The main value of this study will be to inform further research.

PROSPERO registration number

CRD42018087500

For peer review only

For peer review only - http://bmjopen.bmj.com/site/about/guidelines.xhtml

Article Summary

Strengths and limitations of this study

- Prospectively registered review reported consistent with PRISMA guidelines
- This study will provide the first comprehensive review of current evidence on phenotypic subgroupings of atopic dermatitis. This is an important topic in a time of rapid development of new therapeutic options
- It will be difficult to assess bias resulting from non-publication of studies or selective reporting of results
- It may be difficult to synthesize the results, due to expected heterogeneity in identified phenotypes and in the outcomes/other characteristics explored

For peer review only

For peer review only - http://bmjopen.bmj.com/site/about/guidelines.xhtml

Introduction

Atopic dermatitis, also known as atopic eczema, eczema, neurodermatitis and Besnier's prurigo[1, 2], is a complex disease with variable clinical presentations, associations with other atopic diseases and clinical courses.[3] Traditionally atopic dermatitis was characterised as an allergic disease of childhood, but it is now well-established that non-allergic forms exist and patients have been subdivided into those with and without atopy. However, there is much evidence suggesting that this dichotomisation is not clinically useful;[4] despite the use of names such as "atopic dermatitis" or "atopic eczema", up to two thirds of patients with the disease are not atopic and atopy status does not predict outcomes or treatment responses.[5] Furthermore, genetic research in the last decade has led to a paradigm shift in understanding the aetiology of atopic dermatitis, from being considered primarily an allergic/immunological disorder to understanding the additional importance of skin barrier dysfunction.[6, 7] Recent '-omics' research has further highlighted a role of lipid composition and immune pathways in disease pathology and phenotypic presentation.[8-10]

A phenotype is sometimes defined as a set of observable characteristics of an individual resulting from the interaction of its genotype with the environment.[11] This includes, for example, the individual's clinical characteristics, development, and behaviour. In an epidemiological context the word phenotype additionally refers to subtypes of a disease that are defined by different phenotypic appearances. These subtypes may be referred to using a variety of terminology in addition to 'phenotypes': for example, they may be called disease classifications, subgroups, typologies, strata, patterns or taxonomies by different researchers. Subtypes or phenotypes are not to be confused with endotypes, which are sub-types of a disease defined specifically by different functional or pathobiological mechanisms. One endotype could give rise to multiple phenotypes.

Current atopic dermatitis phenotypes are based on clinical (exogenous) features, measureable genetic or immunological (endogenous) features, comorbidities or signs or symptoms course. In general, study participants have been characterised by a particular external characteristic, e.g. history of eczema herpeticum (sometimes called an exophenotype), or internal characteristic, e.g. elevated serum IgE, or low filaggrin protein expression or Th17 activation in skin (endophenotypes). Clinical comorbidities (such as asthma and hay fever or ichthyosis) and symptom trajectories have also been used to define phenotypes. [12-15] Recent studies have highlighted the heterogeneity of signs and symptoms trajectories and have suggested that different clinical courses are associated with immunological characteristics, disease locations and comorbidities such as food allergy.[16, 17]

If there exist a number of atopic dermatitis phenotypes, each of which exhibits homogeneous disease characteristics, this has the potential to inform aetiology (i.e. identify endotypes), patient prognosis and prediction of treatment efficacy. Finding simple, meaningful disease phenotypes can elucidate the different biological pathways underpinning them, leading to the discovery of endotypes (aetiology); knowledge of the markers for these pathways can assist clinicians in diagnosing and managing patients' symptoms more accurately, particularly on whether to expect persistence or remission (prognosis) and whether a potential treatment will work for them (prediction). At present, treatment for long-term atopic dermatitis is not based on disease phenotypes; treatment is symptomatic and may have associated toxicity.[18] The identification of meaningful phenotypes has potential to improve treatment strategies and provide biological insights into the future development of new ones.

No study has systematically reviewed the literature summarizing the currently existing phenotypic classifications of atopic dermatitis. We will do this, aiming to identify previously reported phenotypes in studies specifically designed to identify subtypes of atopic dermatitis. We will also

For peer review only - http://bmjopen.bmj.com/site/about/guidelines.xhtml

describe the features used to define them and whether they were predictive of or associated with any outcomes, concurrent conditions, treatment response or other relevant variables.

Methods and analysis

Eligibility criteria

We will search for studies with any cross-sectional or longitudinal epidemiological or interventional design whose primary or secondary aim is to define or identify subtypes/classifications/phenotypes of atopic dermatitis in humans of any age and gender. Features used to identify phenotypes could be based on either static or dynamic characteristics of their populations, and could include any feature of the disease, including clinical presentation, and genetic, immunological or molecular characteristics. We expect that most studies will contain only individuals with atopic dermatitis but some studies may have included individuals without it, for example as a negative control group[15] or because a formal diagnosis was unavailable. We will include these studies. If included, the control population would be people known to be free of atopic dermatitis or who have a low probability of having it, including people who may have asthma, hay fever and other atopic diseases.

We will exclude: studies of localized eczema such as hand eczema and other types of eczema such as contact dermatitis and adult seborrheic dermatitis (for studies prior to 1990 we may include seborrheic dermatitis of infancy); literature reviews, books, book chapters, case reports, case series and in-progress phenotyping studies (abstracts), but not ongoing birth or other cohort studies; and conference proceedings and abstracts, as they are unlikely to provide sufficient detail on the definitions of atopic dermatitis phenotypes.

Information sources

We will search Ovid Embase, Ovid MEDLINE, and Web of Science from inception to the latest available date at the time of the search for publications in any language using English search terms. We will limit results to human studies published in original journal articles (published or in press, excluding retracted articles). We will interrogate the reference lists from the most recent two major review articles in each database.

Search strategy

We will use conduct the following MEDLINE search:

((exp phenotype/ OR classification OR sub?type OR phenotyp* OR taxonomy OR disease type* OR disease typolog* OR *stratif* OR strata) AND (exp dermatitis, atopic/ OR exp eczema/ OR exp neurodermatitis/ OR eczema* OR atopic dermatitis OR neurodermatitis OR besnier* prurigo))

Searches for the other databases will be matched as closely as possible to this using appropriate syntax and headings.

Study Records

Data management

Literature search results will be uploaded to Covidence web software, which will be used for all stages of the review process including title/abstract screening, full-text screening, data extraction, bias/quality assessment and process flow capture. We will develop and test screening questions

For peer review only - http://bmjopen.bmj.com/site/about/guidelines.xhtml

based on the inclusion and exclusion criteria and a data extraction form based on the outcomes and pilot them on a subset of studies.

Selection process

Two reviewers will scan all titles and abstracts independently. Publications that both reviewers record as ‘not relevant’ will not be retrieved for full-text review; the full text of all others will be retrieved into a ‘short-list’.

Publications will be automatically included in this study if both reviewers independently assess them as meeting the inclusion criteria and excluded from this study where both assess them as not meeting the criteria. Disagreements will be resolved through referral to a third reviewer.

Data collection process

During the short-list review, data from the texts in full will be extracted by two reviewers using a pre-designed data extraction form and disagreements will be resolved by discussion among investigators.

Data Items

We will extract three domains of data from publications included in our study. In the event of subgroup analyses, such as studies reporting different atopic dermatitis phenotypes for males and females, we will extract data from the from the combined-group phenotypes if available. If unavailable, we will extract data for each phenotype separately. We believe it unlikely that more than one subgroup analysis will have been conducted in the absence of a main single analysis, but if this happens we will extract data from the first subgroups reported in the Results section of the paper.

Study data

Design, year(s) conducted, country/countries conducted in, setting conducted in (population-based, specialist etc.), inclusion/exclusion criteria, number of participants, age range of participants, gender balance of participants, notable comorbidities (from study design) including, e.g., proportions with other atopic diseases.

Disease data

Definition codes/criteria, severity definition, prevalence and incidence (if relevant).

Outcomes

A qualitative description of the phenotypes, features (variables) used to define phenotypes, age at the time of phenotype definition, proportion of individuals in each phenotype, qualitative description of any variables statistically significantly associated (in subsequent analyses) with the phenotypes (we will not report the effect estimates), statistical or other method used for classification, whether controls were used in classification algorithm.

Outcomes and prioritisation

The outcomes for this review are atopic dermatitis phenotypes and associated characteristics, all of which are qualitative (i.e. nonnumeric) data.

Primary outcomes are:

For peer review only - http://bmjopen.bmj.com/site/about/guidelines.xhtml

For peer review only

1) atopic dermatitis phenotypes reported in published papers;
2) the features used to define the phenotypes (i.e. the 'exposure' variables); and
3) the characteristics statistically significantly associated (in subsequent analyses) with the phenotypes, if any, which could include: long-term clinical outcomes such as disease persistence or severity; concurrent conditions such as other atopic disease; treatment response; genetic data (e.g. *FLG* mutations); or any other variables reported in studies.

Our secondary outcome is:

1. a brief summary of the methodological approaches used to derive atopic dermatitis phenotypes.

We will not proactively seek particular values of the outcomes described above; we will collect any and all outcomes reported in the publications.

Risk of bias

We will assess quality within and between studies using a checklist modified from the GRADE tool[19] for clinical trials or observational studies. Where relevant, instead of effect estimates and confidence intervals, we will assess equivalent parameters for the methods used to derive subtypes. We will additionally consider upgrading the final assessment by one level if the study is prospective, population-based or has been replicated.

Within studies

We will treat the exposure as the feature(s) used to identify phenotypes, which will vary between studies, and the outcome as the identified phenotypes. We will give an initial rating of 'high quality' for randomized controlled trials or 'low quality' for observational studies and then judge risk of bias based on the extracted information on each of the domains relevant to the study type. Each item will be rated 'high risk of bias' or 'low risk of bias' and will be synthesized according to GRADE recommendations. Based on the synthesized judgement we will consider whether to downgrade or upgrade the initial quality assessment. Final possible quality assessments for individual studies are 'high', 'moderate', 'low' and 'very low'.

Between studies

We anticipate the phenotype descriptions to vary between studies according to the features used to define them (exposure variables), so for a given outcome it may not be possible to synthesize across studies. However, where it is possible we will synthesize study results according to GRADE recommendations. Final possible quality assessments for the body of evidence are 'high', 'moderate' and 'low'

Data Synthesis

The nature of the outcomes for this review preclude quantitative synthesis, so we will report our findings narratively. Our primary outcomes (atopic dermatitis phenotypes, the features used to define them and the outcomes/characteristics associated with them) are all qualitative and we expect them to differ between studies, but where possible we will group them into sensible categories and report frequencies.

For our secondary outcome we will report the type and frequency of methodological approach used to derive the phenotypes.

For peer review only - http://bmjopen.bmj.com/site/about/guidelines.xhtml

For peer review only

We expect heterogeneity in all our outcomes because we have no reason to expect studies will have used similar protocols to explore phenotypes: for example, studies will probably have used different exposure variables to define phenotypes and will represent populations of people with different characteristics. To explore this we will compare the: participant age and gender balance, study design, World Health Organization (WHO) region (Africa, Americas, Southeast Asia, Europe, Eastern Mediterranean and Western Pacific), and disease definition for each study, where possible. We will also look at hospital-based and population-based studies separately.

Meta-biases

Outcomes in this review may be prone to meta-bias resulting from an absence of studies looking at important indicators of phenotypes, non-publication of study results or selective reporting of outcomes. For our qualitative outcomes, it will not be possible to measure this objectively. We will speculate on whether this is likely to be an important limiting factor in interpreting our results.

Patient and Public Involvement

The research questions have been developed in consultation with Dr Sinéad Langan's Senior Clinical fellowship steering committee, which includes patient representation. The authors would like to thank Amanda Roberts for her contributions to discussions.

For peer review only

For peer review only - http://bmjopen.bmj.com/site/about/guidelines.xhtml

Ethics and dissemination

This systematic review protocol was registered with the International Prospective Register of Systematic Reviews (PROSPERO) on 31 January 2018 and was last updated on 5 July 2018. Any amendments to the protocol will be documented on the PROSPERO site contemporaneously, with full explanation of any change. Ethical approval is not required for this study as it is a systematic review. We plan to submit a report of our findings for publication in a peer-reviewed journal. Findings from the report will also support the first author's PhD thesis.

Authors' contributions

AM contributed to the design of the study, developed the search strategy, drafted the PROSPERO protocol and this manuscript and is the guarantor. VA contributed to the design of the study. HW, NP and KA provided critical feedback on the PROSPERO protocol and drafts of the manuscript. DG approved the search strategy. SML contributed to the conception and design of the study, and provided critical feedback on the search strategy, PROSPERO protocol and drafts of the manuscript. All authors read, provided feedback and approved the final manuscript.

Funding statement

Publication of this manuscript was funded by a Wellcome Senior Clinical Fellowship to SML (205039/Z/16/Z). AM and SML were supported by this grant. This research has received funding from the European Research Council under the European Union's Seventh Framework Programme (FP7/2007-2013) / ERC grant agreement no 668954.

Role of the funders

These funders provide salary support. They are not involved in any other aspect of the project, such as the design of the project's protocol and analysis plan. They will not be involved in the collection, analysis and interpretation of data; the writing of the report; or the decision to submit the paper for publication.

Competing interests

The authors declare we have no competing interests.

For peer review only

References

1. Williams HC. Atopic Dermatitis: The epidemiology, causes and prevention of atopic eczema: Cambridge University Press; 2000.
2. Johansson SG, Bieber T, Dahl R, Friedmann PS, Lanier BQ, Lockey RF, et al. Revised nomenclature for allergy for global use: Report of the Nomenclature Review Committee of the World Allergy Organization, October 2003. J Allergy Clin Immunol. 2004 May;113(5):832-6
3. Leung DY, Guttman-Yassky E. Deciphering the complexities of atopic dermatitis: shifting paradigms in treatment approaches. J Allergy Clin Immunol. 2014 Oct;134(4):769-79
4. Williams H, Flohr C. How epidemiology has challenged 3 prevailing concepts about atopic dermatitis. J Allergy Clin Immunol. 2006 Jul;118(1):209-13
5. Flohr C, Johansson SG, Wahlgren CF, Williams H. How atopic is atopic dermatitis? J Allergy Clin Immunol. 2004 Jul;114(1):150-8
6. Sandilands A, O'Regan G, Liao H, Zhao Y, Terron-Kwiatkowski A, Watson R, et al. Prevalent and rare mutations in the gene encoding filaggrin cause ichthyosis vulgaris and predispose individuals to atopic dermatitis. J Invest Dermatol. 2006 Aug;126(8):1770-5
7. Brown SJ, Kroboth K, Sandilands A, Campbell LE, Pohler E, Kezic S, et al. Intragenic copy number variation within filaggrin contributes to the risk of atopic dermatitis with a dose-dependent effect. J Invest Dermatol. 2012 Jan;132(1):98-104
8. Noda S, Suarez-Farinas M, Ungar B, Kim SJ, de Guzman Strong C, Xu H, et al. The Asian atopic dermatitis phenotype combines features of atopic dermatitis and psoriasis with increased TH17 polarization. J Allergy Clin Immunol. 2015 Nov;136(5):1254-64
9. Berdyshev E, Goleva E, Bronova I, Dyjack N, Rios C, Jung J, et al. Lipid abnormalities in atopic skin are driven by type 2 cytokines. JCI Insight. 2018 Feb 22;3(4)
10. Brunner PM, Israel A, Zhang N, Leonard A, Wen HC, Huynh T, et al. Early-onset pediatric atopic dermatitis is characterized by TH2/TH17/TH22-centered inflammation and lipid alterations. J Allergy Clin Immunol. 2018 Jun;141(6):2094-106
11. Oxford English Dictionary. 2018. Phenotype.
12. Beck LA, Boguniewicz M, Hata T, Schneider LC, Hanifin J, Gallo R, et al. Phenotype of atopic dermatitis subjects with a history of eczema herpeticum. J Allergy Clin Immunol. 2009 Aug;124(2):260-9, 9 e1-7
13. Brown SJ, Relton CL, Liao H, Zhao Y, Sandilands A, McLean WHI, et al. Filaggrin haploinsufficiency is highly penetrant and is associated with increased severity of eczema: Further delineation of the skin phenotype in a prospective epidemiological study of 792 school children. British Journal of Dermatology. 2009;161(4):884-9
14. Flohr C, England K, Radulovic S, McLean WHI, Campbell LE, Barker J, et al. Filaggrin loss-of-function mutations are associated with early-onset eczema, eczema severity and transepidermal water loss at 3 months of age. British Journal of Dermatology. 1333;163(6):1333-6
15. Paternoster L, Savenije OEM, Heron J, Evans DM, Vonk JM, Brunekreef B, et al. Identification of atopic dermatitis subgroups in children from 2 longitudinal birth cohorts. J Allergy Clin Immunol. 2017 Nov 10
16. Garmhausen D, Hagemann T, Bieber T, Dimitriou I, Fimmers R, Diepgen T, et al. Characterization of different courses of atopic dermatitis in adolescent and adult patients. Allergy. 2013 Apr;68(4):498-506
17. Roduit C, Frei R, Depner M, Karvonen AM, Renz H, Braun-Fahrlander C, et al. Phenotypes of Atopic Dermatitis Depending on the Timing of Onset and Progression in Childhood. JAMA Pediatr. 2017 Jul 1;171(7):655-62
18. Schmitt J, Schakel K, Schmitt N, Meurer M. Systemic treatment of severe atopic eczema: A systematic review. Acta Dermato Venereologica. 2007;87(2):100-11
19. Guyatt GH, Oxman AD, Vist G, Kunz R, Brozek J, Alonso-Coello P, et al. GRADE guidelines: 4. Rating the quality of evidence--study limitations (risk of bias). J Clin Epidemiol. 2011 Apr;64(4):407-15

For peer review only - http://bmjopen.bmj.com/site/about/guidelines.xhtml

For peer review only

For peer review only - http://bmjopen.bmj.com/site/about/guidelines.xhtml
